# Surgical Treatment of Adolescent Idiopathic Scoliosis with the ApiFix Minimal Invasive Dynamic Correction System—A Preliminary Report of a 24-Month Follow-Up

**DOI:** 10.3390/life13102032

**Published:** 2023-10-09

**Authors:** Susanne Froehlich, Wolfram Mittelmeier, Biren Desai, Subash Jung Pandey, Herbert Raddatz, Bjoern Lembcke, Annett Klinder, Katrin Osmanski-Zenk

**Affiliations:** 1 Orthopedic Clinic and Policlinic, University Rostock Medical Center, 18057 Rostock, Germany; wolfram.mittelmeier@med.uni-rostock.de (W.M.); bjoern.lembcke@med.uni-rostock.de (B.L.); annett.klinder@med.uni-rostock.de (A.K.); katrin.osmanski-zenk@med.uni-rostock.de (K.O.-Z.); 2 Sana Dreifaltigkeits-Hospital Cologne, 50933 Köln, Germany

**Keywords:** adolescent idiopathic scoliosis (AIS), Lenke classification, surgical correction, minimally invasive dynamic correction system

## Abstract

Adolescent idiopathic scoliosis (AIS) is a three-dimensional growth disorder. Corrective surgical procedures are the recommended treatment option for a thoracic angle exceeding 50° and a lumbar major curve of 40°. Over the past few years, dynamic growth modulation implants have been developed as alternatives to permanent fusion. The ApiFix system was designed as a 2D “posterior dynamic device” for curve correction. After implantation in a minimally invasive procedure, it uses polyaxial joints and a self-adjusting rod to preserve the degree of motion and to accommodate the patient’s growth. It provides an effective method of controlling deformity and fills the gap between the conservative treatment of major curves that are >35° and the fusion procedure. The objective of the two-center cohort study was the analysis of the correction results of patients, who underwent surgical intervention with the ApiFix system. The inclusion criteria were AIS, Lenke type 1 or type 5, a major curve on bending films of ≤30°, and an angle of the major curve of between 35° and 60°. Postoperative radiograph data were obtained longitudinally for up to 24 months of follow-up and compared to preoperative (preop) values. For comparisons of the different time points, non-parametric tests (Wilcoxon) or paired *t*-tests for normally distributed values were used to analyze repeated measures. Overall, 36 patients (25 female and 11 male) were treated with the ApiFix system from April 2018 to October 2020. Lenke type 1 was identified in 21 (58%) cases and Lenke type 5 was identified in 15 (42%) cases. The average angle of the thoracic major curve for Lenke 1 was 43°. The preoperative lumbar major curve (Lenke 5) was determined to be 43°. Over a follow-up of 24 months, a correction of the major curve to an average of 20° was observed for Lenke 1 and that to an average of 15° was observed for Lenke 5. Lenke type 1 and type 5 showed significant changes in the major curve over the individual test intervals in the paired comparisons compared to the starting angle (Lenke 1: preop—24 months, 0.002; Lenke 5: preop—24 months, 0.043). Overall, 11 events were recorded in the follow-up period, that required revision surgery. We distinguished between repeated interventions required after reaching the maximum distraction length of the implant due to the continued growth of the patient (n = 4) and complications, such as infections or problems associated with the anchorage of the implant (n = 7). The results from the present cohort revealed a statistically significant improvement in the postoperatively measured angles of the major and minor curves in the follow-up after 24 months. Consequently, the results were comparable to those of the already established vertebral body tethering method. Alignment in AIS via dynamic correction systems in combination with a possible growth modulation has been a treatment alternative to surgical fusing procedures for more than a decade. However, the long-term corrective effect has to be validated in further studies.

## 1. Introduction

Adolescent idiopathic scoliosis (AIS) is characterized as a persistent curvature of the spine in the coronal plane with a deformity greater than 10° in association with a rotation of the vertebra. The extent of deformity is assessed via radiographic imaging in the upright or standing position. The Cobb technique represents the most commonly used diagnostic tool for the identification of scoliosis [1,2,3,4].

The progression of AIS is influenced by the age of the patient at the beginning of the treatment, menarche and the Risser stage [5]. An onset of AIS at an earlier age in combination with a large major curve is more likely to be associated with a higher risk of progression of the deformity due to the expected adolescent peak in growth. The importance of a consistent classification of AIS was postulated early on to make reliable decisions on treatment approaches [6,7]. Especially, the Lenke classification was developed to provide a comprehensive and reliable system to categorize all surgical AIS curves [8]. For this classification system, two-dimensional radiographic images of the upright coronal and sagittal planes as well as supine side bending radiographs are analyzed with regard to curve type (1–6, Table 1), a lumbar spine modifier (A = center sacral vertical line between pedicles, B = center sacral vertical line touches apical bodies, and C = center sacral vertical line completely medial), and a sagittal thoracic modifier (- = below normal, N = normal, and + = above normal); thus, each curve is defined by three individual components. The analysis includes the three regions of the radiographic coronal and sagittal planes, as curve types are based on location. The proximal thoracic, main thoracic, and thoracolumbar/lumbar curves are classified to be either the major curve or minor curves. The minor curves are additionally categorized as structural and nonstructural types. According to Lenke et al., the major and structural minor curves should be included in instrumentation and fusion, while leaving the nonstructural minor curves unfused [8,9]. 

The Society of Scoliosis Orthopedic Rehabilitation and Treatment (SOSORT) developed guidelines for the treatment of patients with scoliosis (observation, physical therapy, bracing and surgery) based on the current scientific evidence [3,7]. For example, the guidelines on conservative treatment that were passed in the Consensus Session of the first combined SOSORT/IRSSD Meeting in May 2016 were published 2018 and included 68 recommendations in total for treatment approaches. AIS treatment is primarily determined via the major curve. Scoliosis patients with major curves of less than 20° usually receive clinical check-ups in regular intervals, every 3–6 months, and radiological check-ups in case of suspected progression. Scoliosis-specific physical therapy is part of its “management” despite the limited evidence that it prevents the progression of AIS [10,11,12]. For patients with curves of larger than 25°, the most common treatment option of fulltime bracing is recommended in AIS during growth. However, for curves with smaller angles of 20–30°, braces should only be prescribed when a progression of 5° or more occurs between consecutive visits [3]. The therapeutic approach of bracing in scoliosis is the only non-surgical treatment for which there is a high level of scientific evidence (level of evidence I, strength of recommendation A [7]). Besides “traditional” daytime or fulltime braces, treatment with the Bending Nighttime Brace, which aims at overcorrection during the night, has become an established approach in bracing [1,2,11,12]. 

Corrective surgery is indicated at a major thoracic curve of 50° and higher and a lumbar curve of 40° [1,2]. Current recommendations to achieve the correction of deformity and prevent scoliotic curve progression is permanent spinal fusion. While instrumented fusion achieves reliable long-term outcomes [13], major concerns are the cessation of natural growth and reduced motion [4]. Alternatively, fusionless procedures were developed to address these issues. Dynamic systems use a variety of surgical approaches to achieve curve correction while preserving the residual growth capacity and mobility of the spine. The vertebral body stapling (VBS) method uses a complex anterolateral approach, which is similar in principle to temporary epiphysiodesis for the axial correction of the lower extremities [14]. The staples are anchored in the vertebra on the convex side of the spine which reduces the pressure on the concave side and is intended to result in the axis-aligned growth of the spine. The system is used especially in children with stage 0–1 Risser. A major drawback is the limited success in curve correction with thoracic curves of over 35°. Nowadays, this surgical procedure is rarely performed. Vertebral body tethering (VBT) represents an advancement from VBS; however, the principle used to correct deformity as well as its specific operative access are still the same as those in VBS [15]. In VBT, screws are inserted laterally into the vertebrae and connected with a flexible cord (tether) [14,16,17]. VBT is most commonly recommended for a single major thoracic curve with a nonstructural lumbar and proximal thoracic curve [18]. A recent literature review by Raitio et al. identified the ideal candidates for the VBT procedure to be patients with a suitable amount of remaining growth (Sanders stage 3 to 5), flexible right thoracic curves (40 to 60°, bending less than 30°) and a rib hump of less than 20° [18]. The most common reported complications with VBT were pulmonary and instrumentation-related adverse events (tether breakage; overcorrection) [18,19,20,21]. 

**Figure 1:**
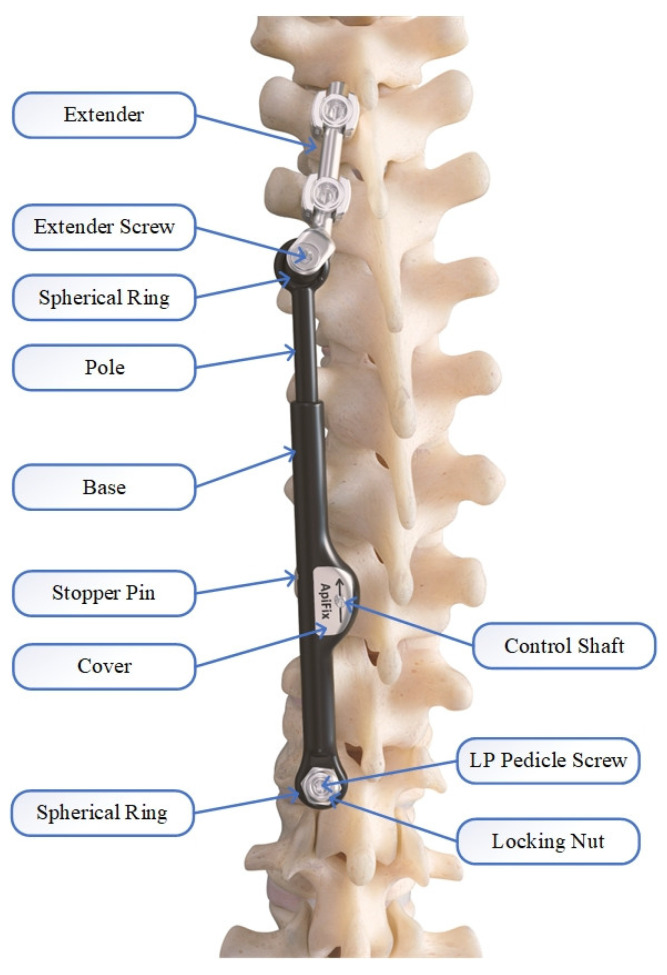
Schematic diagram of the ApiFix construct; Source: company OrthoPediatrics.

While VBS and VBT represent compression-based techniques, other dynamic systems, including the ApiFix system (Figure 1), use distraction-based techniques in the fusionless surgical treatment of AIS [14]. The ApiFix system was designed as a posterior dynamic device for single curves which, in contrast to fusion instrumentations, permits the movement of the curve itself [17,22,23]. As the design is only intended for the correction of single curves, the system was solely approved for Lenke type 1 and 5 (Table 1), although it is recommended for skeletally immature as well as mature patients (Risser 0–5).

**Figure 2:**
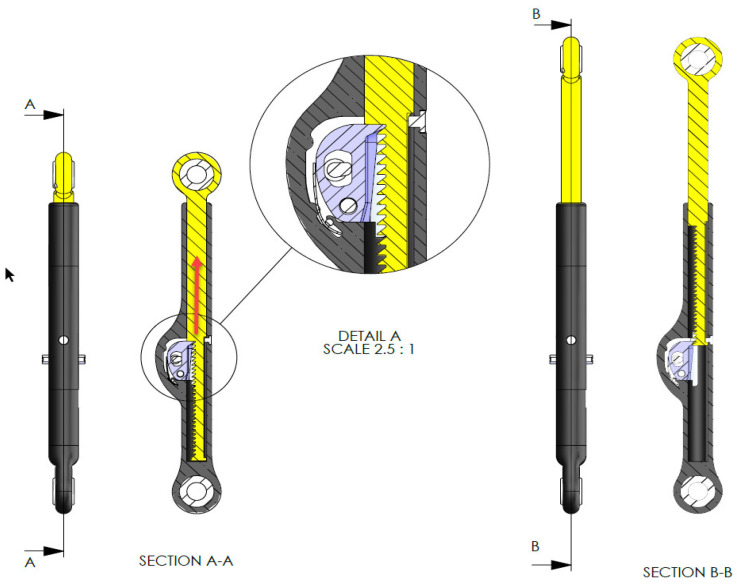
Schematic image of the ApiFix device with an amorphous diamond-like ceramic coat (coated areas are represented by the dark gray areas) and a ratchet mechanism (section A-A and detail A) to allow distraction of up to a maximum of 50 mm. Source: company OrthoPediatrics.

Curve correction and growth modulation is accomplished via incremental ratchet lengthening. During tensile load, e.g., when the patient performs the recommended exercises, the ratchet mechanisms elongate and this elongation is then locked via a locking tooth under compressive load. The locking tooth rotates around a 2 mm pin and interacts with the toothed area to allow only a unidirectional movement and is pressed down by a flat spring to prevent posterior sliding (Figure 2).

**Figure 3:**
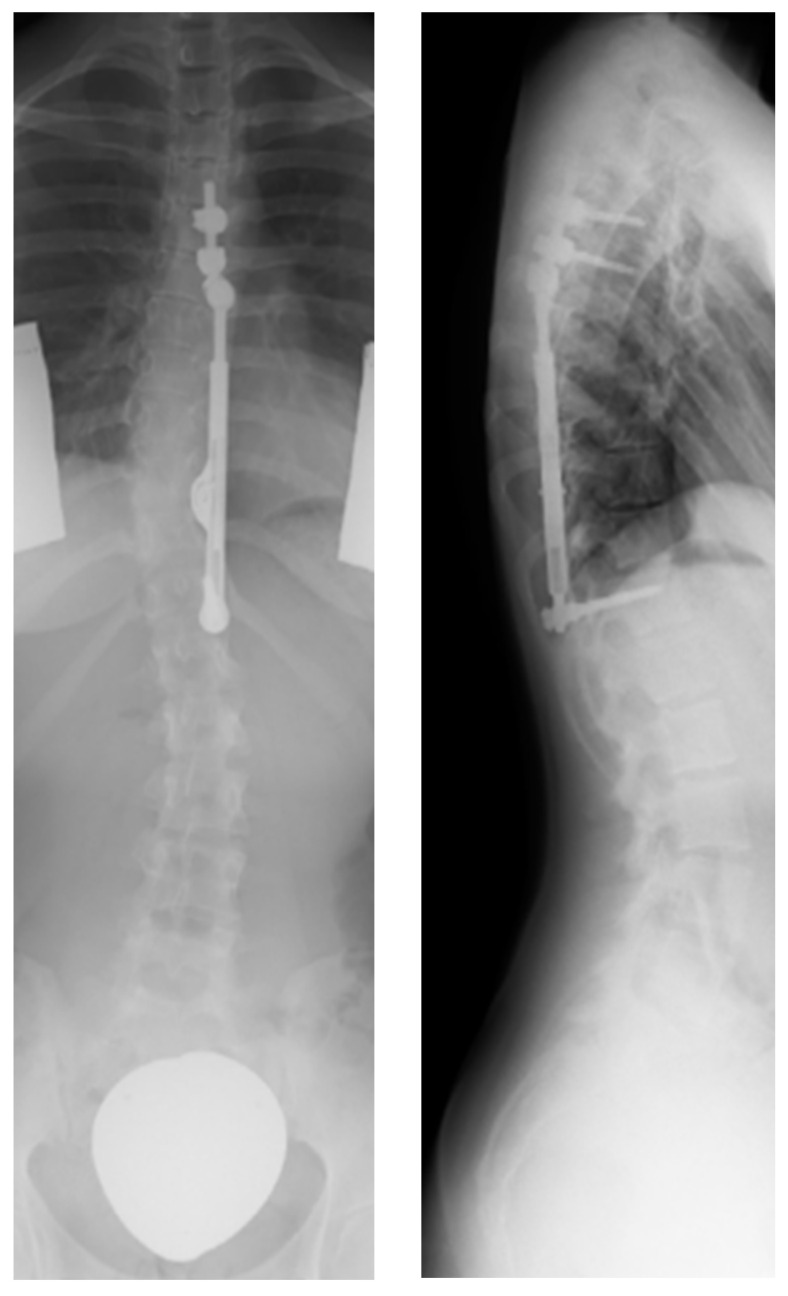
ApiFix with extender 24 months postoperatively.

All parts including the pedicle screws are made of titanium alloy (Ti-6AL-4V), while the outside of the implant as well as the screws are coated with amorphous diamond-like ceramics (ADLC). The coating is intended to minimize friction and wear. The length of the ratchet changes with increasing implant length. The shortest ApiFix implant is 85 mm long with a distraction reserve of 30 mm for elongation, while the longest is the 125 mm and reaches a maximum elongation of 50 mm. The pedicle screws are placed on the concave side of the curve. To reduce the load on the implant caused by biomechanical stress, a special extender was introduced to anchor the superior end of the implant with two screws instead of just one (Figure 3) [17,22,23]. This was carried out to divert the maximum force away from the lower screw. For this, it was necessary to position the extender at an angle of 10–15° (Kite angle) to the C7 plumb line of the spine. 

The implantation of the ApiFix device is less invasive than other procedures and involves fewer instrumented segments [22]. Initially, the aim was to achieve scoliotic curve correction via a gradual elongation through the ratchet mechanism in combination with physical therapy, while intraoperatively, only some mild pre-distraction was carried out [22,23]. However, after the analysis of the initial results with the implant, it became apparent that intraoperatively mild distraction must be performed at initial insertion. Thus, at the end of the surgical procedure a singular extension of the adjustable rod is performed to attain the required distraction. In patients without further expected growth (Risser 4 or 5), standardized physical therapy exercises can still lead to additional rod elongation postoperatively. For patients who are skeletally immature, the capacity for spinal growth is furthermore supposed to have a positive influence on the growth of the vertebral bodies themselves (growth modulation). Therefore, the ApiFix device is considered to be a so-called “internal brace”, i.e., a device that allows growth control until skeletal maturity is reached and the possibility of subsequent removal after the cessation of growth without a consecutive loss of the correction [11,14]. After surgical treatment, no external brace is necessary. Moderate sporting activities (not competitive sports) after the procedure are allowed. 

ApiFix has been approved by the US Food and Drug Administration (FDA) since 2021 [24]. The device received the Conformité Européenne mark (CE) in 2013 [25]. Since the device was only approved recently, post-approval data on the long-term benefits of ApiFix system are still pending. A case series with 3 patients followed for up to 24 months and a cohort study with 45 patients with two to four years of follow-up reported significant scoliotic curve reduction in all patients and growth modulation in skeletally immature patients [22,23]. However, another study with 20 patients was terminated prematurely due to a high rate of adverse events. While this study also observed significant curve correction, there was a lack of postoperative distraction [26]. The aim of our cohort study was to observe the longitudinal changes in curve correction after the implantation of the improved ApiFix system, which utilizes three pedicle screws and facilitates distraction intraoperatively. This retrospective study included all patients, who were treated with the ApiFix system between 2018 and 2020 in the two study centers. Changes in major and minor curves as well as kyphosis were recorded at defined time points for up to 24 months to assess the extent of curve correction and the progression of AIS. 

## 2. Materials and Methods

### 2.1. Study Design

**Figure 4:**
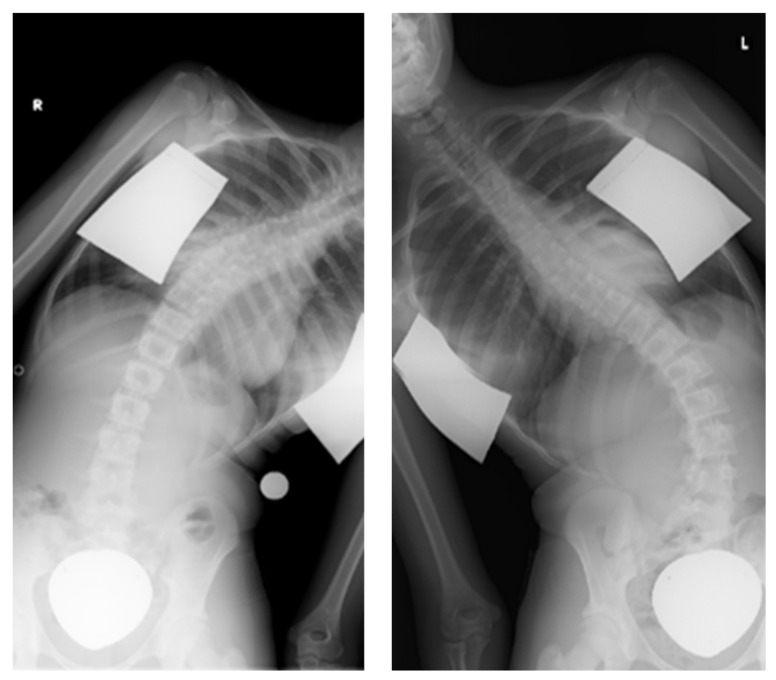
Preoperative major curve bending radiographs.

In this retrospective study, all patients who underwent the ApiFix procedure between April 2018 and October 2020 at two spine centers, namely the Sana Dreifaltigkeits-Hospital in Cologne and the Orthopedic Clinic and Policlinic of the University Medical Center in Rostock, were included in the analysis. The study was approved by the Ethics Committee of the University Rostock Medical Center (number: A 2020–0294). The eligibility of patients to undergo the ApiFix procedure was assessed in accordance with the indications and contraindications listed in the FDA approval [24]. Briefly, the indications were AIS with a single major curve of 35–60°, an angle of ≤30° on lateral side bending radiographs (Figure 4) and Lenke type 1 and type 5, while intra-spinal malformations/deformities, segmentation disorders, a lateral bending curve > 30°, a major curve > 60° and secondary scoliosis were contraindicative [22,23]. Risser 5 was not an exclusion criterion.

Patients underwent preoperative magnetic resonance imaging of the complete spine to rule out intraspinal deformities. Preoperative radiographs were evaluated for each patient with the Cobb technique to measure the curve pattern, and curves were categorized in accordance with the Lenke classification. All patients received an ApiFix implant with the optional extender, i.e., with three pedicle screws. Intraoperatively, the required distraction was performed manually with a specialist tool provided by the manufacturer of the implant immediately after the implantation of the ApiFix device. The extent of intraoperative distraction was adjusted individually based on the curve pattern of the patient and was determined by the operating surgeon. After wound closure, a dose of 2 g of Vancomycin was applied in situ to prevent wound infection. This procedure, ensuring aseptic conditions, has been used routinely in one center and was later adopted by the other center. Six weeks after surgery, patients started with standardized physical therapy in their place of residence. Patients were followed after surgery for up to 24 months to assess curve correction and the progression of AIS. 

### 2.2. Outcome Measures

The primary endpoint was the assessment of longitudinal changes in scoliotic curve correction which was based on measurements of the major curve, minor curve and sagittal thoracic modifier (kyphosis) at 6 weeks, 6 months, 12 months and 24 months after surgery. At discharge, only the major curve was determined. Recorded patient characteristics included age, gender, Risser stage, the onset of menarche and prior conservative AIS treatment. Additionally, implant length, the duration of surgery and intraoperative and postoperative complications were documented. 

### 2.3. Data Analysis and Statistical Evaluation

If not stated otherwise, data are reported as absolute numbers with percentages in brackets for nominal values and as mean ± standard deviations (SDs) for metric values. Longitudinal changes were visualized using GraphPad Prism 9 (GraphPad Software, San Diego, CA, USA), while statistical analysis was performed with the statistics software program SPSS 27.0 (IBM Deutschland GmbH, Ehningen, Germany). Only pseudonymized data were analyzed.

The normal distribution of the data was assessed with a Shapiro–Wilk test. Due to the longitudinal nature of the measurements, comparisons of curve angles between all individual time points were performed each with individual paired *t*-tests for normally distributed data and with a Wilcoxon test if data were not normally distributed. Differences between time points were considered significant for *p* > 0.05. 

## 3. Results

In total, 36 ApiFix devices were implanted in the two study centers from April 2018 to October 2020. Twenty-seven of these interventions were performed in the specialist orthopedic clinic in Cologne and nine were performed in the Orthopedic Clinic and Policlinic in Rostock.

During the study period, 25 (69%) female patients and 11 (31%) male patients with an average age of 15.1 ± 1.7 years were treated. At the time of surgical indication, approximately half of the patients had received bracing as a form of prior conservative therapy including 10 patients who had been treated with a fulltime brace, six adolescents with Bending Nighttime Brace and three boys with a daytime and nighttime orthosis brace. Preoperative assessment identified 21 patients (58%) as Lenke type 1 and 15 patients (42%) as Lenke type 5. The skeletal maturity of the patients was categorized according to the Risser stage. A detailed list of the number of patients in each Risser stage is shown in Table 2. At the time of surgery, two thirds of patients had more or less reached skeletal maturity with 25 of 36 patients (69%) being categorized as Risser stage 4 and 5. In 22 patients (88%), menstrual bleeding had already occurred. No patients with Risser 0–1 were treated with ApiFix in the two study centers.

During primary surgery, 15 patients (42%) received a 105 mm long implant, 20 patients (56%) received a 125 mm long implant, and one patient (3%) received a 115 mm implant. The average surgery time was 125 ± 29 min.

### 3.1. Curve Correction during Follow-Up

**Figure 5:**
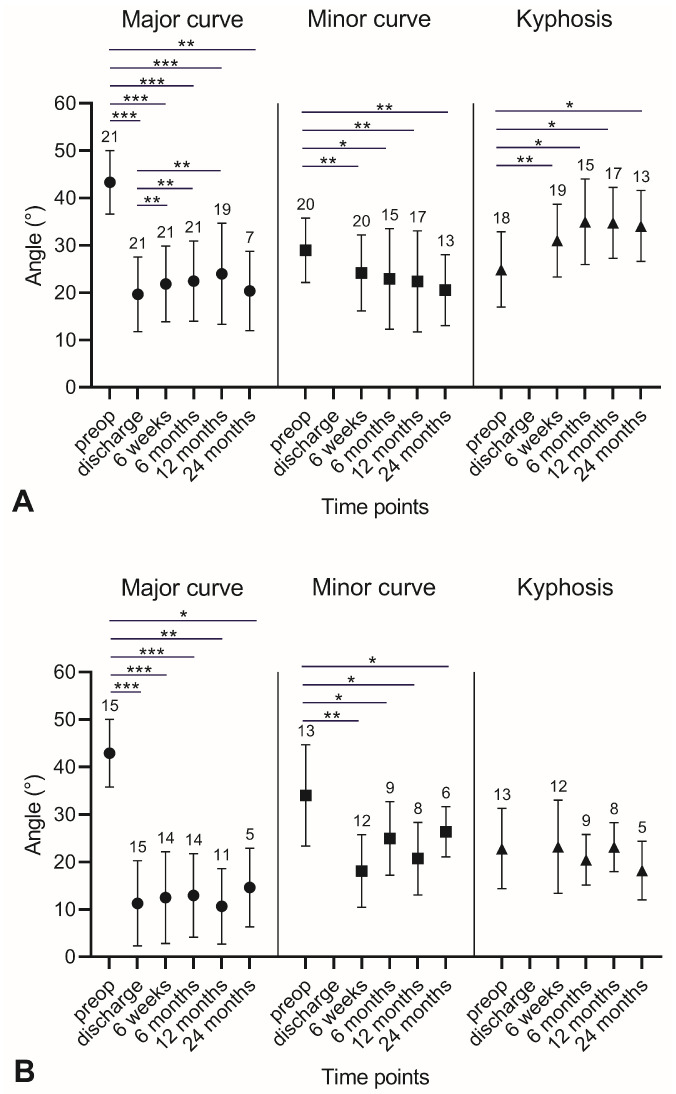
Major curve, minor curve and kyphosis in Lenke type 1 (**A**) & Lenke type 5 (**B**) patients at the preoperative (preop) and the different postoperative time points. Data are presented as mean values with standard deviation & sample size. Statistical analysis was performed with either individual paired *t*-tests (**A**) or individual Wilcoxon tests: * *p* < 0.05; ** *p* < 0.01; *** *p* < 0.001.

Treatment with ApiFix resulted in a significant correction of deformity. The average preoperative thoracic major curve (Lenke 1) of 43 ± 7° was significantly reduced to 20 ± 8° at the last follow-up at 24 months (*p* = 0.002, paired *t*-test). For patients with Lenke type 5, the improvement was even more pronounced. The preoperative lumbar curve (Lenke 5) of 43 ± 7° decreased to 15 ± 8° at 24 months (*p* = 0.043, Wilcoxon test). Similar effects between preoperative and 24-month values were observed for the minor curves (Lenke type 1: 29 ± 7° to 20 ± 8°, *p* = 0.005, as determined via paired *t*-tests; Lenke type 5: 34 ± 11° to 26 ± 5°, *p* = 0.028, as determined via Wilcoxon tests). The time course for major curves, minor curves and kyphosis is shown in Figure 5. The graphs show that correction was achieved through the surgical intervention and was then maintained for the entire follow-up period. There was no further postoperative distraction. However, there was a slight, but significant deterioration in the curve correction of the major curve after discharge in Lenke type 1 patients. No further deterioration was noticed at later time points. In Lenke type 5 patients, no effect after discharge was observed. The improvement in curve correction for Lenke type 1 was associated with an increase in the sagittal thoracic modifier (kyphosis) (Figure 5).

### 3.2. Complications/Revisions

**Figure 6:**
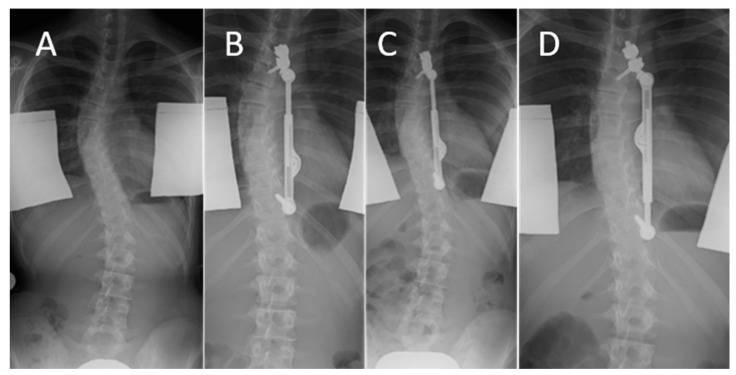
Follow-up of an 115 mm ApiFix implant. Preoperative radiograph (**A**), initial ApiFix device after implantation (**B**) and with a fully utilized distraction length 22 months after index surgery (**C**), and replacement with a 125 mm ApiFix up-side down system (**D**).

All recorded events in the study were implant-associated. No neurological deficits or pulmonary abnormalities were observed in the follow-up period. In our cohort of 36 patients, revision was required in 11 cases (31%) during the 24-month follow-up period. Among these cases were four patients, who outgrew their implant. An example is shown in Figure 6. The complete distraction length was reached as a consequence of a growth spurt, so further curve correction as well as the maintenance of the correction result was not possible.

In all four patients, the initial device was replaced with a longer ApiFix implant, thus continuing the treatment with the ApiFix system. According to the ApiFix procedure, intraoperative distraction was executed again for the replacement implant. This resulted in an additional improvement of the major curve in these patients (Figure 6D). The time after which the maximum distraction of the device due to continued growth was reached in the individual patients is listed in Table 3.

The other seven revisions (19%) were due to various reasons, these mainly being infections (n = 3) and problems associated with the anchorage of the implant. Table 4 shows an overview of complications and the reasons for revision and the adopted measures. 

## 4. Discussion

In the past decade, dynamic deformity correction has received more interest as a surgical treatment approach in AIS [14,17]. While fusion procedures provide sustainable long-term outcomes, the associated reduced spinal mobility can only be addressed via the use of dynamic procedures [18]. Dynamic systems limit the progression of scoliotic deformity by mechanically restraining the spine during the remaining spinal growth period [18,28]. However, the available data for this regarding long-term success are limited [14,17,29,30]. At present, predominantly, VBT and ApiFix are used as dynamic treatments for AIS [31]. According to the available literature, both approaches showed similar results for the correction of the scoliotic curve [22,23,29]. However, considerably more data are available on the long-term outcome of VBT than there are for the ApiFix system [15,16,17,18,19,20,21,22,23,24].

The first ApiFix publication in 2015 by Floman et al. [22] mainly described the system, including the underlying principle for curve correction, and documented its application in a case series of three patients. The procedure including results was described as follows: instrumentation in 3–4 levels, a surgery time of less than 60 min, an incision length of 10 cm, 50 mL blood loss, and a deformity correction of 46% of the initial major curve. Only the treatment of Lenke type 1 was performed and no implant failure was observed in the follow-up periods of at least 6 months to a maximum of 24 months. The average curve correction was stated to be 25°–35°. No complications associated with the surgical treatment or the implant were reported [22]. The results of curve correction were confirmed in a subsequent publication by Floman et al. [23] in 2021. Depending on skeletal maturity, deformity improved significantly after surgery, reaching a correction of up to 55% of the initial curve (Risser 0–1, from 47.6° to 26.4°; Risser 2–3, from 46° to 20.4°; and Risser 4–5, from 41.5° to 26.2°) at the final follow-up at 2–4 years. The differences between the groups regarding curve correction were not significant. The analysis of our cohort showed a comparable improvement in the major and minor curves in the coronal radiographic view as well as in the sagittal profile in the follow-up period of 24 months. At the final follow-up, a deformity correction of the major curve of up to 53% was achieved in patients with Lenke type 1, while that of Lenke type 5 patients improved on average by 65%. The Lenke type 1 correction was also associated with an improvement in kyphosis. In contrast, a study by Stadhouder et al. [26] reported considerably less improvement with average curve corrections from 45.4° preoperatively to 31.0° postoperatively. Since the extent of improvements seems to depend on skeletal maturity, the reason for the different results might be due to the status of the patients at the time of surgery. Thus, the analysis of the study population in its entirety without considering skeletal maturity represents a crucial limitation of our study. However, since there were no patients with Risser 0–1 and only less than a third of immature patients (Risser 2–3), we decided against subdividing the small group of 36 patients even further in the statistical analysis. Furthermore, growth modulation was not assessed in our study, since Floman et al. showed that apical wedging as a parameter for growth modulation was only significantly reduced in patients with Risser 0–1 [23]. 

Apart from the benefits in AIS, the risks for the patient of invasive treatment have to be considered when adapting a new procedure. While Floman et al. [23] documented only four revision surgeries in the 45 patients analyzed, a study by University Medical Center Amsterdam presented high rates of revision associated with the surgery and the implant [26]. Ten of twenty patients had to be revised as a consequence of serious complications, with seven of them already having been revised during the first year. The study described six osteolyses around the screws, two rod breakages, one failure of the ratchet mechanism and one case with persisting pain for no explainable reason. Metallosis was observed in all revision patients. *Cutibacterium acnes* was detected in six cases after microbiological testing. Due to the high rate of complications (50%), the study was terminated early by the research group [26]. 

When analyzing the required surgical interventions in the follow-up period of our study, we had to distinguish between expected and unexpected events. The rate of revisions within 24 months in our study was 31%. However, four revisions were due to growth spurts, which can be expected in skeletally immature patients. In these patients, the maximum distraction of the implant was reached within the follow-up period and the insertion of a longer implant was necessary for the continuation of curve correction. The requirement of a longer implant may even be considered a positive result of dynamic correction, since it indicates that the remaining growth capacity was not impaired by the ApiFix implant. Here, the therapeutic approach of an “internal brace” in the sense of a growth modulation constitutes an essential part of the treatment. The replacement is relatively straightforward, since ApiFix can be disconnected intraoperatively without problems. A replacement of the distal screw is necessary, while the extender remains in place. Compared to other distraction-based techniques such as growing rods, which in a prospective study by Kocyigit et al. [32] required 12 lengthening procedures on average, a single adjustment for growth as observed in these four patients might be an advantage of the ApiFix system due to the self-adjusting rod. Even in conservative scoliosis treatment, every external brace must be remade as a result of the growth of the patient. After growth is finished, external braces can be trained off. In this respect, it is quite conceivable that the implant could be removed after growth is completed, possibly without a significant loss of curve correction. 

Thus, when excluding implant replacements due to growth modulation, the rate of revisions as a consequence of implant-associated complications was 19%. While this rate is still higher than the 9% reported by Floman et al. [23], it is considerably lower than the 50% complication rate observed with ApiFix in the study by Stadhouder et al. [26]. However, it is similar to the revision rate of 13.1% and 15% in VBT as reported in two recent systematic reviews on VBT [18,29]. A common problem in VBT is pulmonary complications [19,33]. The present study did not observe any neurological or pulmonary postoperative deficits in the cohort. It can be assumed that the dorsal approach compared to VBT reduces comorbidities induced by the surgery.

Another common complication in VBT is tether breakage. The systematic review by Zhang et al. [29] reported confirmed or suspected broken tethers in 21.3% of cases. However, a breakage of the tether does not necessarily lead to a loss of correction. Baroncini et al. [34] described in a study that of 152 curves treated with VBT, 12 cases experienced “tether breakage” within 6 months postoperatively, and another 37 experienced this within one year, and a further 35 experienced this after 24 months. The result of this study was that tether breakage occurring during the first 12 months postoperatively led to a significant loss of the correction of the major curve. Mechanical complications (11%), such as a loosening of screws or an extender set being too short (kite angle) were also a main cause of revision in our study. However, the rate of these was much lower than the 45% rate reported by Stadhouder et al. [26] and we also did not observe osteolysis in these cases. The histological examination showed no sign of metallosis or any indication of the presence of metal wear particles. The difference might be due to the improved design of the implant. Stadhouder et al. [26] suggested for the manufacturer to strengthen the proximal construct with two pedicle screws instead of one as they attributed their high failure rate and osteolysis to the excessive load on the single screws during the movement of the spine. This suggestion has been implemented and the currently available implant uses three pedicle screws. However, we still noticed black-colored tissue during the revisions. This was due to the wear of the ceramic diamond coating which occurs as the ApiFix is still a dynamic implant. Until now, the reaction of pleural tissue to the wear products of the coating is unknown. In summary, it should be noted that dynamic correction systems are exposed to substantial biomechanical loads that are triggered by growth itself as well as by muscles and ligaments due to stress [21].

All surgical interventions carry a risk of infection. We recorded three infections (8%). After the administration of 2 g of Vancomycin in situ as a standard in both study centers, no further infections were observed in the later treatments. Similarly to what was found by Stadhouder et al. [26], *Cutibacterium acnes* was detected as the causative pathogen in two of the three infections. However, our overall infection rate was much lower and is consistent with the infection rate of 7% described in a recent retrospective study on infection after ApiFix procedures [35].

The present study has a few limitations. The measurement results of the presented radiological parameters were gathered by a single surgeon in each study center. Thus, no “four-eye-principle” was applied. The presented cohort is small and did not allow a subdivision to be made in the analysis of skeletal immature and mature patients with regard to the outcome. The follow-up period was only 24 months. 

Our results show that the dynamic correction of AIS with the ApiFix system in combination with possible growth control during skeletal immaturity is a good treatment alternative compared to fusion surgeries and the VBT system, provided that the patients are carefully selected with regard to the treatment criteria. Risser 4 and 5 are not contraindications for the ApiFix procedure. The aspect of growth modulation does not necessarily play a role in the treatment of these cases, but the limited instrumentation and the dynamics of the implant are to be seen as an advantage. However, it has to be noted that according to Hacquebord and Leopold [27] only Risser 5 is an accurate indicator of vertebral growth cessation. Therefore, since the majority of our skeletally mature patients were still at Risser 4, some growth modulation might have occurred. 

Despite the promising results of our study, it has to be stated that these results are only preliminary, since they are based on a relatively small cohort and follow-up did not extend beyond 2 years. Further studies are necessary to evaluate the long-term outcomes of using the ApiFix implant. Furthermore, the loss of curve correction after implant removal has to be investigated.

## Figures and Tables

**Table 1:** Classification of curve type according to Lenke [8].

Curve Type	Structural Curve/Curves	Nonstructural Curve/Curves	No. of Structural Curves
1 Main thoracic	Main thoracic #	Proximal thoracic Thoracolumbar/lumbar	1
2 Double thoraric	Main thoracic # Proximal thoracic	Thoracolumbar/lumbar	2
3 Double major	Main thoracic # Thoracolumbar/lumbar	Proximal thoracic	2
4 Triple major	Main thoracic # Thoracolumbar/lumbar Proximal thoracic		3
5 Thoracolumbar/lumbar	Thoracolumbar/lumbar #	Main thoracic Proximal thoracic	1
6 Thoracolumbar/lumbar-main thoracic	Thoracolumbar/lumbar # Main thoracic	Proximal thoracic	2

# = major curve.

**Table 2:** Skeletal maturity of patients according to Risser classification [27].

Risser Stage	No. of Patients	Percentage
2	4	11.1
3	7	19.4
4	17	47.2
5	8	22.2

**Table 3:** Repeated interventions required after reaching the maximum distraction length of the implant due to the continued growth of the patient.

Time after Index Surgery	Interventions
13 months	Maximum distraction distance reached after growth spurt, implant replacement with longer implant including renewed intraoperative distraction
18 months	Maximum distraction distance reached after growth spurt, implant replacement with longer implant including renewed intraoperative distraction
20 months	Maximum distraction distance reached after growth spurt, implant replacement with longer implant including renewed intraoperative distraction
22 months	Maximum distraction distance reached after growth spurt, implant replacement with longer implant including renewed intraoperative distraction

**Table 4:** Complications within the first 24 months.

Time after Index Surgery	Reason for Revision	Action Taken
7 days	Distal ApiFix screw was too long	Replacement with a shorter screw
3 months	Screw dislocation with lateralization of the implant	Implant replacement
11 months	Scar tissue excision with subsequent infection and fistula on the caudal wound pole	Complete removal of implant
16 months	Loosening of screws with loss of curve correction	Complete removal of implant
19 months	Loss of curve correction	Correction of kite angle, implant replacement with longer implant including renewed intraoperative distraction
21 months	Low-grade infection with Cutibacterium acnes	Complete removal of implant
22 months	Low-grade infection with Cutibacterium acnes	Complete removal of implant

## Data Availability

The data presented in this study are available on request from the corresponding author. The data are not publicly available, but can be obtained from the Department of Clinical Research at the Orthopedic Department of the University Medicine Rostock if required.
